# ViralCC retrieves complete viral genomes and virus-host pairs from metagenomic Hi-C data

**DOI:** 10.1038/s41467-023-35945-y

**Published:** 2023-01-31

**Authors:** Yuxuan Du, Jed A. Fuhrman, Fengzhu Sun

**Affiliations:** 1 Department of Quantitative and Computational Biology, University of Southern California, Los Angeles, CA USA; 2 Department of Biological Sciences, University of Southern California, Los Angeles, CA USA

**Keywords:** Metagenomics, Computational models

## Abstract

The introduction of high-throughput chromosome conformation capture (Hi-C) into metagenomics enables reconstructing high-quality metagenome-assembled genomes (MAGs) from microbial communities. Despite recent advances in recovering eukaryotic, bacterial, and archaeal genomes using Hi-C contact maps, few of Hi-C-based methods are designed to retrieve viral genomes. Here we introduce ViralCC, a publicly available tool to recover complete viral genomes and detect virus-host pairs using Hi-C data. Compared to other Hi-C-based methods, ViralCC leverages the virus-host proximity structure as a complementary information source for the Hi-C interactions. Using mock and real metagenomic Hi-C datasets from several different microbial ecosystems, including the human gut, cow fecal, and wastewater, we demonstrate that ViralCC outperforms existing Hi-C-based binning methods as well as state-of-the-art tools specifically dedicated to metagenomic viral binning. ViralCC can also reveal the taxonomic structure of viruses and virus-host pairs in microbial communities. When applied to a real wastewater metagenomic Hi-C dataset, ViralCC constructs a phage-host network, which is further validated using CRISPR spacer analyses. ViralCC is an open-source pipeline available at https://github.com/dyxstat/ViralCC.

## Abstract (web summary)

Metagenomic Hi-C enables genome retrieval in microbial samples. Here, the authors develop an integrative method to recover complete viral genomes and detect virus-host pairs using metagenomic Hi-C data.

## Introduction

Viruses constitute the most divergent and ubiquitous biological organism on earth with an estimated global abundance of 10^31^ ^1^. Viruses have enormous impacts on ecosystems as predators and/or parasites within microbial communities through the lysogenic or lytic cycle infecting bacteria and archaea^2,3^. For instance, viruses contribute significantly to the biogeochemical cycling of carbon and nitrogen in aquatic habitats^4,5^ and are implicated in certain diseases such as inflammatory bowel disease and severe acute malnutrition in human systems^6,7^. Therefore, the interest in viromics has risen dramatically in the past two decades.

Since the number of viruses that can be traditionally cultivated in the laboratory is too limited to assess viral diversity^8^, metagenomics, as a culture-independent sampling strategy, has been widely exploited to recover viral genomes and to identify the hosts of these newly discovered viruses, one of the most difficult aspects of studying viruses in microbial communities^9–11^. Metagenomic whole genome shotgun sequencing (WGS) directly extracts genomic fragments from various environmental samples, generating a large number of short reads that are subsequently assembled into contigs^12–14^. Metagenomic viral contigs are then identified from large assemblies based on sequence composition, sequence similarity, and/or detection of viral proteins^15–17^. However, viral genome assembly from shotgun reads is challenging^18^ and short viral contigs may only represent segments of entire viral genomes^19^. Incomplete viral fragments have a significantly adverse impact on the downstream analyses, including the characterization of the underlying viral diversity and abundance, prediction of host and functional capacity^20,21^. Therefore, metagenomic viral binning, defined as a process to group viral contigs from the same species into viral metagenome-assembled genomes (vMAGs), is valuable, especially for giant viruses^22^.

Most of traditional shotgun-based binning tools are developed to recover eukaryotic, bacterial, and archaeal genomes^23–26^ and ignore the challenges associated with viruses, such as the lack of universal single-copy genes and relatively small size of viral genomes. Additionally, those binning tools exploiting microbial marker gene analysis are not applicable for viruses^24,27,28^. CoCoNet^29^ and vRhyme^30^ are two existing methods specifically dedicated to metagenomic viral binning. CoCoNet trains a neural network using both composition and co-occurrence features of viral contigs across samples to predict the probability that two viral contigs originate from the same genome. vRhyme utilizes single- or multisample coverage effect size comparisons to calculate coverage differences between viral contigs. To process the sequence composition information, vRhyme first pretrains supervised machine-learning-based classification models using genome fragments. Then, the nucleotide feature similarity vector between two viral contigs is input into the classification models to predict the probability value that viral contigs originate from the same genome. Finally, vRhyme constructs a weighted network, where each node is a viral contig and an edge weight is calculated by dividing the coverage difference by the probability value. Networks are further refined into vMAGs. However, both CoCoNet and vRhyme may be critically impaired when there are not enough samples to construct reliable co-abundance profiles of viral contigs, i.e., profiles showing which contigs share consistent abundance values across multiple samples and are therefore likely to come from the same genome.

Metagenomic high-throughput chromosome conformation capture (metagenomic Hi-C) has been developed in recent years to simultaneously recover metagenome-assembled genomes (MAGs) and determine virus-host pairs from a single microbial community sample^31–37^. Combined with the conventional shotgun sequencing, metagenomic Hi-C applies a genomic proximity ligation technique to construct chimeric junctions between metagenomic sequences in close proximity within the same cell. After sequencing, millions of Hi-C read pairs are generated and subsequently aligned to contigs assembled from the shotgun reads. Contigs belonging to the same genome display enriched Hi-C contact frequencies compared to those from different genomes^31^, resulting in dozens of nearly complete bacterial genomes retrieved by publicly available Hi-C-based binning tools, such as MetaTOR, bin3C, and HiCBin^38–40^. Although recovering high-quality viral genomes is vital and prerequisite for downstream analyses, apart from a proprietary and commercial genome reconstruction service called ProxiPhage^41^, Hi-C-based binning methods with open-source pipelines are not developed to retrieve viral genomes. For example, HiCBin requires the taxonomic annotation of some contigs by TAXAassign (https://github.com/umerijaz/TAXAassign) to generate the intra-species contacts in the normalization step^42^ while TAXAassign can hardly annotate viral contigs, resulting in the inability of HiCBin to bin viral contigs.

In addition to the difficulties in recovering vMAGs, tools for benchmarking the performance of viral genome retrieval remains rare in metagenomic Hi-C experiments. CheckV has been widely used to estimate the completeness of vMAGs by comparing them to a large database curated from NCBI GenBank and environmental samples^43^. However, unlike the CheckM which takes advantage of universal single-copy marker genes to assess both completeness and contamination of prokaryotic MAGs^44^, CheckV is unable to estimate the contamination of vMAGs since there is no such marker gene set available for viruses^21^. CheckV is also limited in its ability to assess the completion of vMAGs since randomly grouping two viral contigs together generally increases completion. Moreover, though methods based on simulating known viral contigs from NCBI RefSeq viral genomes^45^ have already been employed to estimate the binning results of shotgun-based methods^29,30^, they cannot be generalized to evaluate Hi-C-based binning approaches since few studies have been conducted on modeling Hi-C interactions for viral contigs. Therefore, it is imperative to design a systematic and comprehensive benchmarking strategy for Hi-C-based metagenomic viral binning.

**Fig. 1:**
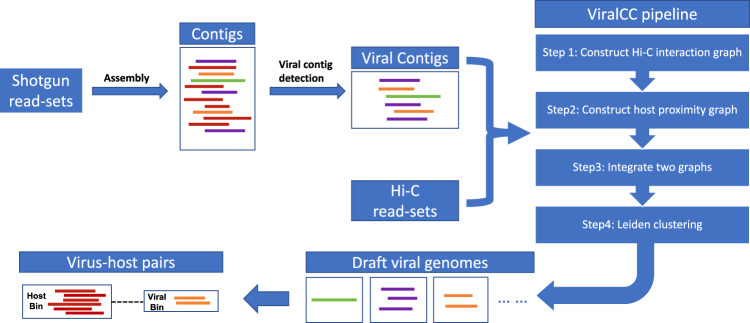
Overview of the ViralCC pipeline. The general workflow of ViralCC to retrieve high-quality viral genomes and determine virus-host pairs. Shotgun reads are first assembled into contigs, to which Hi-C paired-end reads are aligned. Viral contigs are subsequently identified. Leveraging Hi-C linkages and the virus-host proximity structure to link viral contigs, ViralCC constructs the Hi-C interaction graph and the host proximity graph. After integrating two graphs, ViralCC employs Leiden clustering to reconstruct draft viral genomes, and additionally detects the virus-host pairs based on recovered viral genomes and Hi-C linkages.

To tackle the problem of a paucity of viral binning methods in metagenomic Hi-C experiments, we developed ViralCC, a Hi-C-based binning method dedicated to recovering complete viral genomes and determining virus-host pairs. The general pipeline of ViralCC is shown in Fig. 1. ViralCC not only considers the Hi-C interaction graph, but also puts forward a host proximity graph of viral contigs as a complementary source of information to the Hi-C interaction map. Two graphs are then integrated together, followed by Leiden graph clustering^46^, to generate draft viral genomes. We compared ViralCC to VAMB^26^, CoCoNet^29^, vRhyme^30^, MetaTOR^38^, and bin3C^39^. Our experiments indicated that ViralCC substantially improved the CheckV completeness of viral genomic bins on real metagenomic Hi-C datasets. Moreover, we put forward a systematic strategy to benchmark the viral genome retrieval performance in metagenomic Hi-C experiments by generating mock metagenomic Hi-C datasets from real samples. The ground truth of all mock viral contigs is known in mock datasets while Hi-C interactions between mock viral contigs can be obtained directly from real samples without simulation. Leveraging mock metagenomic Hi-C datasets derived from three real samples, we further demonstrated that ViralCC outperformed other binning methods and recovered viral genomes with higher completeness and lower contamination. Finally, we showed that the virus-host pairs can be determined based on the recovered viral genomes.

## Results

### Generating mock metagenomic Hi-C datasets for benchmarking

All viral contigs detected by VirSorter were assessed by CheckV to select single contigs with high completeness as putative reference genomes. As a result, 51 putative reference genomes, with length ranging from 11,410 bp to 194,784 bp were generated from the human dataset; 11 putative reference genomes from 11,452 bp to 42,000 bp were obtained from the cow fecal dataset; and 17 putative reference genomes, ranging from 11,455 bp to 127,910 bp were derived from the wastewater dataset (Supplementary Table 1).

We then constructed mock viral contigs by splitting the putative viral genomes and obtained 1010, 94, and 279 fragmented mock viral contigs from the three datasets, respectively (Supplementary Table 1). For each real metagenomic Hi-C dataset, mock viral contigs were mixed with all non-viral contigs (i.e., contigs that are not identified as viral contigs by VirSorter), followed by the alignment of Hi-C paired-end reads to construct the mock metagenomic Hi-C dataset. The analyses of binning mock viral contigs on the mock human gut dataset were presented in the main text. We also provided benchmarking results on the mock wastewater and the mock cow fecal datasets in the Supplementary Note 1.

### Integrating the Hi-C interaction graph and the host proximity graph improves binning performance on the mock human gut dataset

We first constructed the Hi-C interaction graph ${{{{{{{{\mathcal{G}}}}}}}}}_{{{{{{{{\rm{hic}}}}}}}}}$ and the host proximity graph ${{{{{{{{\mathcal{G}}}}}}}}}_{{{{{{{{\rm{host}}}}}}}}}$ for 1010 mock viral contigs from the mock human gut dataset. There are 2699 edges in ${{{{{{{{\mathcal{G}}}}}}}}}_{{{{{{{{\rm{hic}}}}}}}}}$. The parameter *k* for ${{{{{{{{\mathcal{G}}}}}}}}}_{{{{{{{{\rm{host}}}}}}}}}$ was tuned to be 30, which means that any two viral contig nodes with an edge in ${{{{{{{{\mathcal{G}}}}}}}}}_{{{{{{{{\rm{host}}}}}}}}}$ were linked to at least the same 30 host contigs by the Hi-C interaction. This resulted in 2698 edges in ${{{{{{{{\mathcal{G}}}}}}}}}_{{{{{{{{\rm{host}}}}}}}}}$. Among these 2698 edges in ${{{{{{{{\mathcal{G}}}}}}}}}_{{{{{{{{\rm{host}}}}}}}}}$, 14.5% of the edges were spurious edges, which were defined as the edges that linked two contigs from different putative reference genomes in ${{{{{{{{\mathcal{G}}}}}}}}}_{{{{{{{{\rm{host}}}}}}}}}$. We then integrated ${{{{{{{{\mathcal{G}}}}}}}}}_{{{{{{{{\rm{host}}}}}}}}}$ and ${{{{{{{{\mathcal{G}}}}}}}}}_{{{{{{{{\rm{hic}}}}}}}}}$ into ${{{{{{{{\mathcal{G}}}}}}}}}_{{{{{{{{\rm{int}}}}}}}}}$, which contained 4397 edges. We could observe 1000 common edges between ${{{{{{{{\mathcal{G}}}}}}}}}_{{{{{{{{\rm{host}}}}}}}}}$ and ${{{{{{{{\mathcal{G}}}}}}}}}_{{{{{{{{\rm{hic}}}}}}}}}$, accounting for around 37% of the total number of edges in either graph.

We applied the Leiden clustering on ${{{{{{{{\mathcal{G}}}}}}}}}_{{{{{{{{\rm{hic}}}}}}}}}$, ${{{{{{{{\mathcal{G}}}}}}}}}_{{{{{{{{\rm{host}}}}}}}}}$, and ${{{{{{{{\mathcal{G}}}}}}}}}_{{{{{{{{\rm{int}}}}}}}}}$, respectively, and assessed the binning results using four clustering metrics: F-score, ARI, NMI, and homogeneity (Supplementary Table 2). ${{{{{{{{\mathcal{G}}}}}}}}}_{{{{{{{{\rm{int}}}}}}}}}$ outperformed both ${{{{{{{{\mathcal{G}}}}}}}}}_{{{{{{{{\rm{hic}}}}}}}}}$ and ${{{{{{{{\mathcal{G}}}}}}}}}_{{{{{{{{\rm{host}}}}}}}}}$ in terms of all four clustering metrics. We also evaluated the completeness and contamination of each vMAG (Supplementary Table 3). Specifically, 8 near-complete, 3 substantially complete, and 5 moderately complete vMAGs were recovered based only on ${{{{{{{{\mathcal{G}}}}}}}}}_{{{{{{{{\rm{hic}}}}}}}}}$, while 12 near-complete and 2 substantially complete vMAGs were retrieved based only on ${{{{{{{{\mathcal{G}}}}}}}}}_{{{{{{{{\rm{host}}}}}}}}}$. In contrast, employing the integrative graph ${{{{{{{{\mathcal{G}}}}}}}}}_{{{{{{{{\rm{int}}}}}}}}}$ for clustering could reconstruct 26 near-complete, 2 substantially complete, and 4 moderately complete vMAGs. The improvement of binning performance by integrating two graphs indicated the Hi-C interaction graph and the host proximity graph were complementary to each other on binning viral contigs.

### ViralCC outperforms other binning methods on the mock human gut dataset

ViralCC was compared to VAMB, CoCoNet, vRhyme, bin3C, and MetaTOR on the mock human gut dataset (see Methods). VAMB is a general shotgun-based binning tool while bin3C and MetaTOR are general Hi-C-based binning pipelines. CoCoNet and vRhyme are two shotgun-based binning methods specifically designed for clustering sequenced viral particles.

**Fig. 2:**
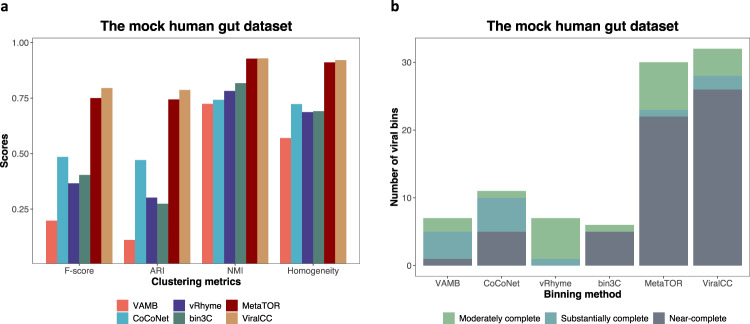
ViralCC outperforms other binning methods on the mock human gut dataset. Comparison of viral genome retrieval performance according to (**a**) clustering metrics and (**b**) completeness and contamination criteria (Moderately complete: 50% ≤ completeness <70%, contamination ≤ 10%; Substantially complete: 70% ≤ completeness <90%, contamination ≤ 10%; Near-complete: completeness ≥ 90%, contamination ≤ 10%). ViralCC outperforms other binning methods on the mock human gut dataset. Source data are provided as a Source Data file.

As shown in Fig. 2a, VAMB, CoCoNet, vRhyme, bin3C, and MetaTOR achieved 0.198, 0.485, 0.366, 0.404, and 0.750 in terms of F-score, respectively, which was improved to 0.795 by ViralCC. The ARI scores for viral bins produced by VAMB, CoCoNet, vRhyme, bin3C, and MetaTOR were 0.111, 0.471, 0.302, 0.274, and 0.744. In contrast, ViralCC increased the ARI score to 0.787. As for the NMI, VAMB, CoCoNet, vRhyme, bin3C, and MetaTOR obtained 0.724, 0.742, 0.782, 0.817, and 0.928, whereas ViralCC achieved a score of 0.929. ViralCC also improved the homogeneity score to 0.921 from 0.570, 0.723, 0.687, 0.691, and 0.911, achieved by VAMB, CoCoNet, vRhyme, bin3C, and MetaTOR, respectively.

VAMB, CoCoNet, vRhyme, bin3C, and MetaTOR could recover 1, 5, 0, 5, and 22 near-complete vMAGs, respectively, while ViralCC increased this number to 26 (Fig. 2b). In total, ViralCC could retrieve 32 high-quality vMAGs out of 51 reference genomes whereas VAMB, CoCoNet, vRhyme, bin3C, and MetaTOR could reconstruct 7, 11, 7, 6, and 30 high-quality vMAGs, respectively. Moreover, we also found that ViralCC had a better performance than other binners in recovering near-complete vMAGs from large putative viral genomes (Supplementary Note 2). Altogether, ViralCC outperformed other binning methods as it recovered viral genomes with higher completeness and lower contamination based on the mock metagenomic Hi-C dataset. Notably, MetaTOR and ViralCC were comparable according to the NMI and the homogeneity scores, indicating that both approaches could recover high-purity viral contig bins. On the other hand, ViralCC achieved better performance than MetaTOR in terms of F-score and ARI (Fig. 2a) while retrieving more complete bins (Fig. 2b) from the mock metagenomic Hi-C dataset. This shows the effectiveness of combining host proximity information with Hi-C interaction information.

### Binning analyses of viral contigs on three real metagenomic Hi-C datasets

VirSorter detected 791, 1338, and 2757 viral contigs from the human gut, cow fecal, and wastewater samples, respectively. Viral contigs were binned using different methods for the three datasets. The CheckV completeness of viral bins was estimated to evaluate the binning quality. We referred to viral bins with CheckV completeness above 90% as draft viral genomes with high completion and denoted bins with CheckV completeness above 50% as draft viral genomes with medium completion.

**Fig. 3:**
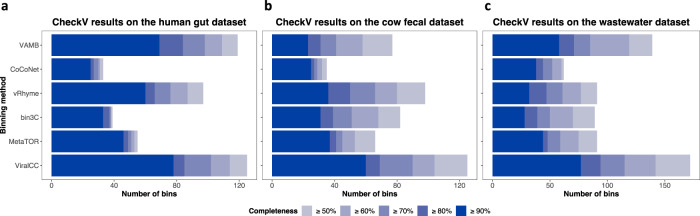
ViralCC outperforms other binners on real metagenomic Hi-C datasets. Comparison of draft viral bins retrieved by different binning tools according to the CheckV completeness standard on the (**a**) human gut, (**b**) cow fecal, and (**c**) wastewater datasets. ViralCC can retrieve more complete viral genomes compared to VAMB, CoCoNet, vRhyme, bin3C, and MetaTOR from all three real metagenomic Hi-C samples. Source data are provided as a Source Data file.

For the human gut dataset, ViralCC identified 465 viral bins with sizes ranging from 3001 bp to 307,395 bp, and yielded more high and medium-completion draft viral genomes than any other tested methods (Fig. 3a). For the cow fecal dataset, ViralCC constructed 574 viral bins with sizes ranging from 3002 bp to 157,462 bp. It generated substantially more medium and high-completion draft viral genomes than other methods, specifically exceeding the numbers of high-completion draft genomes from VAMB, CoCoNet, vRhyme, bin3C, and MetaTOR by 161%, 140%, 66.7%, 93.5%, and 62.1%, respectively (Fig. 3b). From the wastewater dataset, ViralCC established 1240 viral bins with sizes ranging from 3006 bp to 461,626 bp, and could reconstruct 32.8%, 103%, 141%, 175%, and 75% more high completion draft genomes compared with VAMB, CoCoNet, vRhyme, bin3C, and MetaTOR, respectively (Fig. 3c). ViralCC also recovered markedly more draft viral genomes with medium completion.

**Fig. 4:**
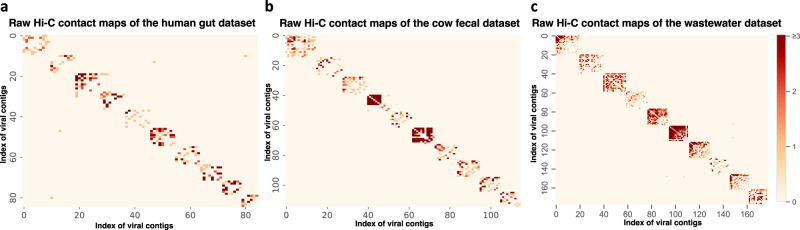
Heatmaps of raw Hi-C contact matrices of the top ten vMAGs from real metagenomic Hi-C datasets. Heatmaps of raw Hi-C contact matrices of the top ten vMAGs from the (**a**) human gut, (**b**) cow fecal, and (**c**) wastewater datasets with the contig index as the axis unit. The vMAGs were first ranked by their numbers of contigs and then the contigs within each vMAG were ranked by their sizes. The scale bar shows the number of raw Hi-C contacts between viral contigs.

Altogether, the analyses of three real metagenomic Hi-C datasets demonstrated that ViralCC retrieved more complete viral genomes compared to VAMB, CoCoNet, vRhyme, bin3C, and MetaTOR, which was consistent with our observations from the mock metagenomic Hi-C datasets. We also constructed a random binning model based on the configuration random graph^47^ as control experiments (Supplementary Note 3). The model randomly assigned edges to match the degree sequence of viral contigs in the integrative graph. ViralCC outperformed the random control according to the CheckV completeness criteria. Moreover, we sorted vMAGs by the number of viral contigs in descending order. If multiple vMAGs contained the same number of viral contigs, they were further sorted by the bin size in descending order. Contigs in each vMAG were also sorted by the contig length in descending order. We then plotted the raw Hi-C contact maps (see Methods) of the top ten vMAGs for the three datasets with either the contig index (Fig. 4) or the contig size (Supplementary Fig. 1) as the axis unit, respectively, which confirmed the valid reconstruction of the viral genomes. The specific number of viral contigs and the bin size of these vMAGs are shown in Supplementary Tables 4 to 6.

Finally, we explored the relationships between the quality of Hi-C datasets and the vMAG retrieval performance. The 3D ratio and the qc3C CI were employed to measure the quality of Hi-C datasets (see Methods). Specifically, the 3D ratios were 23.3%, 38.3%, and 54.9% for the human gut, cow fecal, and wastewater datasets, respectively (Supplementary Table 7). The midpoints of the qc3C CI for the three datasets were 5.938%, 52.07%, and 30.66%, respectively (Supplementary Table 7). Though the higher 3D ratio does not necessarily mean more informative linkages between contigs^36^, we still observed that compared to the traditional shotgun-based binning methods, the improvement of binning performance by ViralCC was remarkable on metagenomic datasets with high-quality Hi-C libraries.

### Annotation of vMAGs demonstrated the high purity of the vMAGs at the family level

We annotated 191, 320, and 693 vMAGs in total at the family level for the human gut, cow fecal, and wastewater datasets, respectively. We found that 173 (90.6%) out of 191 vMAGs in the human gut sample, 265 (82.8%) out of 320 vMAGs in the cow fecal sample, and 592 (85.4%) out of 693 vMAGs in the wastewater sample contained only viral contigs from the same family, demonstrating the high purity of vMAGs at the family level.

**Fig. 5:**
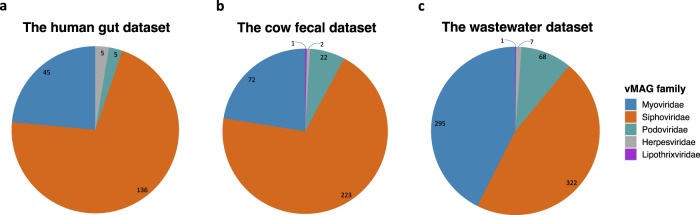
Taxonomy statistics of annotated vMAGs from real metagenomic Hi-C datasets. Taxonomy statistics of annotated vMAGs on the (**a**) human gut, (**b**) cow fecal, and (**c**) wastewater datasets. The numbers on the graph indicate the number of vMAGs belonging to different families. Source data are provided as a Source Data file.

As shown in Fig. 5, the vMAGs were dominated by tailed bacteriophages of the order *Caudovirales* and vMAGs belonging to the families *Myoviridae*, *Siphoviridae*, and *Podoviridae* were found in all three samples^48^. Bacteriophages, mainly *Siphoviridae*, dominated the two gut samples^49^. Compared to the other samples that were more dominated by *Siphoviridae*, *Myoviridae* and *Siphoviridae* vMAGs were of similar abundance in the wastewater sample, as reported for water environments^50–53^.

### Phage-host network in the wastewater sample

We discovered virus-host pairs based on the vMAGs recovered by ViralCC, and showed the results from the wastewater dataset in the main text below. The results of virus-host detection from the human gut and cow fecal datasets are shown in the Supplementary Notes 4 and 5.

**Fig. 6:**
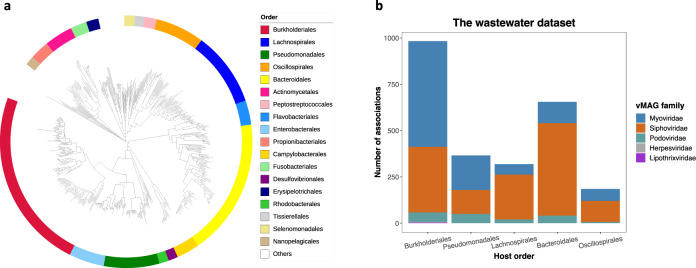
Taxonomic annotations of MAGs and the apparent infection spectrum of vMAGs from the domestic wastewater sample. (**a**) Taxonomic annotations of MAGs recovered by HiCBin from the domestic wastewater sample. *Burkholderiales*, *Pseudomonadales*, *Lachnospirales*, *Bacteroidales*, and *Oscillospirales* were the predominant orders. (**b**) The apparent infection spectrum of vMAGs from the wastewater sample. vMAGs belonging to the family *Myoviridae* mainly targeted hosts from the order *Burkholderiales* and a large number of vMAGs from the family *Siphoviridae* could infect *Bacteroidales* bacteria. Source data are provided as a Source Data file.

For non-viral contigs, expected to be largely bacterial, HiCBin generated 1253 MAGs, which were assessed by CheckM (v1.1.3, parameter: lineage wf)^44^. The quality evaluation results are shown in Supplementary Table 8. Among 1253 MAGs, 600 MAGs could be unambiguously annotated by GTDB-TK^54^ and the taxonomy classification results were visualized using ITOL^55^ (Fig. 6a). *Burkholderiales*, *Pseudomonadales*, *Lachnospirales*, *Bacteroidales*, and *Oscillospirales* were the predominant orders in the wastewater sample. *Burkholderiales* and *Pseudomonadales* were common orders reported in water environments^56,57^. *Lachnospirales*, *Bacteroidales*, and *Oscillospirales* were reported in the gut microbiomes^58^; these are reasonable to be detected in this domestic wastewater sample from around 25,000 people^57^.

A total of 1065 (85%) out of 1253 MAGs were associated with at least one viral MAG. We then explored the infection spectrum of annotated vMAGs on hosts from different orders (Fig. 6b). We observed that vMAGs from the family *Myoviridae* mainly targeted hosts from the order *Burkholderiales*, which is consistent with previous findings that some phages belonging to the family *Myoviridae* could lyse bacteria from *Burkholderia*^59^. A large number of vMAGs belonging to the family *Siphoviridae* could infect *Bacteroidales* bacteria^60^. Moreover, we unexpectedly observed that 4 vMAGs apparently infecting members of the order *Burkholderiales* came from the family *Herpesviridae*, which previously has been reported only to infect animals, including human-beings^61^. Further research is needed to determine if these reveal a true infection or if the proximity ligation occurred in a non-infection situation (e.g. extracellularly).

### Validate virus-host pairs using CRISPR spacer analysis on the wastewater dataset

We predicted the CRISPR spacers in host MAGs using PILER-CR (v1.06)^62^ and 925 CRISPR spacers were detected. Then, we aligned these spacers to vMAGs using BLAST^63^ with parameters ‘-task blastn-short -evalue 1e-5’. The alignments with bitscore below 45 were further filtered out^36^. In this way, 16 robust hits between host MAGs and virus MAGs were found using CRISPR spacer analysis.

Among those 16 hits, 13 virus-host MAG pairs (81.3%) were also associated by the Hi-C linkages. Noticeably, according to CRISPR spacer analysis, we observed that vMAG 1198 (family: *Siphoviridae*) was associated with two host MAGs from the *Fusobacteriales* order while these two host MAGs were the only two associated hosts of vMAG 1198 predicted by the Hi-C interactions.

### Running time of ViralCC

ViralCC was executed on one computing node of a 2.40 GHz Intel Xeon Processor E5-2665 with 50,000 MB RAM provided by the Advanced Research Computing platform at University of Southern California. ViralCC consumed 22.5 min, 76.6 min, and 21.7 min running time on the human gut, cow fecal, and wastewater samples, respectively.

## Discussion

ViralCC is an open-source Hi-C-based binning method for viral genome retrieval. Unlike other Hi-C-based binning tools using only Hi-C contact maps. ViralCC exploits a host proximity graph based on the virus-host proximity structure as a supplementary source of connections between viral contigs. We demonstrate that ViralCC outperformed other tools on real metagenomic Hi-C datasets according to the CheckV completeness criteria. Notably, considering that randomly binning viral contigs into vMAG does not reduce the CheckV completeness compared to the completeness of each of the individual contigs, it is necessary to construct a random binning model as control experiments when the CheckV completeness is used as the evaluation metric. Moreover, we observe that the improvement of binning performance by ViralCC was significant from metagenomic datasets with high-quality Hi-C libraries compared to the shotgun-based binning methods, indicating the potential importance of good-quality Hi-C libraries on viral genome retrieval.

Since the assessment by CheckV software is not comprehensive, we put forward a systematic benchmarking strategy to assess the performance of binning viral contigs using mock metagenomic Hi-C datasets. We expect that this benchmarking strategy can facilitate the evaluation of any Hi-C-based binning tools in viral genome retrieval studies. However, there are also limitations and biases in the benchmarking strategy. Since we only choose viral genomes that can be recovered by a single contig from the whole community, our benchmarking method inevitably underestimates the true diversity of the virus community. The effectiveness of the benchmarking is also less convincing if there are few putative viral genomes. Moreover, though we have shown the low fraction of spurious contacts in the host proximity graph using the mock metagenomic Hi-C datasets, we cannot obtain the results from the real datasets because it is challenging to know the true labels of viral contigs from the real datasets. Finally, we observe that the sizes of putative viral genomes tend to be small in the benchmarking method (Supplementary Note 6). Though all pipelines are treated equally on the same set of mock viral contigs derived from the selected putative viral genomes, the sizes of putative viral genomes should be accounted for in the benchmarking considering that the full recovery of a larger putative viral genome requires a binner to correctly group more viral contigs into a single bin from the mock datasets.

Apart from the direct binning of viral contigs as we discussed here, training a classification model to distinguish confidently labelled viral bins and bacterial bins can also contribute to providing a highly enriched candidate set of viral bins from bulk metagenome data^64^. Viral genome retrieval, combined with the Hi-C proximity ligation also sheds light on the infection mechanisms and unveils entirely active virus-host interactions.

Compared to a popular approach, CRISPR spacer analysis, which can reflect historic linkages between viruses and hosts^65,66^, metagenomic Hi-C experiments are able to detect active virus-host pairs at a single time point. Chen et al.^67^ used metagenomic Hi-C experiments to validate virus-host associated pairs predicted by CRISPR in activated sludge (AS) samples using Illumina sequencing and Nanopore sequencing separately. They validated 11 out of 21 and 16 out of 28 virus-host associated pairs predicted by CRISPR based on the Illumina and combined Illumina/Nanopore sequenced samples, respectively, leveraging Hi-C linkages. In our study, we validated 13 out of 16, 3 out of 4, and 2 out of 2 virus-host pairs predicted by CRISPR based on the wastewater, human gut, and cow fecal datasets, respectively (see Results, Supplementary Notes 4 and 5). Both studies clearly show how analyses of metagenomic Hi-C data can be a powerful tool in recovering virus-host pairs that are otherwise difficult to determine (e.g. from non-cultured organisms). It should be noted that some CRISPR-predicted virus-host associations indicate historical associations that may not be present in a given sample, and such pairs cannot be detected by Hi-C^67^. And it must also be kept in mind that some virus-bacteria associations apparent from proximity ligation might be a result of proximity of bacterial and viral DNA from a mechanism other than infection; thus, unexpected results like our reported apparent herpesvirus infection of *Burkholderiales* should be validated before jumping to extraordinary conclusions.

In the future, it will be interesting to explore whether existing binning methods can resolve closely related viruses residing in the same bacterial host based on virus-host proximities. Moreover, recent studies have found that specific viruses have mechanisms enabling multiple viral genomes to infect the same host cell, which is called the co-infection^68^. Leveraging the Hi-C proximity ligation to discover the existence of co-infection for multiple phages within the same cell is another potential topic for future research.

## Methods

### Real metagenomic Hi-C datasets

Three real metagenomic Hi-C datasets, all previously published, were employed to validate the performance of viral genome retrieval and to discover virus-host pairs. Experiments from the previously published papers are briefly repeated here.

#### The human gut dataset

This dataset was derived from the microbiome of a human gut and was composed of one WGS library (NCBI accession: SRR6131123) and two separate Hi-C libraries constructed by two four-cutter restriction enzymes, MluCI and Sau3AI (NCBI accession: SRR6131122 and SRR6131124)^34^. The Illumina HiSeqX Ten was used to sequence the shotgun and Hi-C libraries, creating 151 bp paired-end reads. The two Hi-C libraries consisted of 48.8 million (MluCI library) and 41.7 million (Sau3AI library) read pairs, respectively. The sequencing of the raw WGS library produced 250.9 million read pairs (ratio Hi-C:shotgun = 0.36).

#### The cow fecal dataset

The cow fecal sample was collected and processed at the Beef and Sheep Research Centre of Scotland’s Rural College^69^, generating one shotgun library (NCBI accession: ERX2333418) and two Hi-C libraries fragmented using either the Sau3AI or MluCI restriction enzymes (NCBI accession: ERX2548555 and ERX2548556). After sequencing all libraries by the Illumina HiSeqX platform at 150 bp, 159.5 million paired-end reads were obtained in the shotgun library while the two Hi-C libraries contained 86.2 million (Sau3AI library) and 59.3 million (MluCI library) paired-end reads, respectively (ratio Hi-C:shotgun = 0.91).

#### The wastewater dataset

In the wastewater (WW) sample^57^, the shotgun library (NCBI accession: SRR8239393) was prepared using the DNeasy PowerWater kit while the Hi-C library (NCBI accession: SRR8239392) was produced by a proprietary Hi-C preparation kit (Phase Genomics, Inc). The cutting enzymes utilized in the experiment were Sau3AI and MluCI. All read-sets were sequenced by the HiSeq 4000 at the length of 150 bp. There were 269.3 million and 95.3 million paired-end reads for the WW shotgun metagenomic and Hi-C read-sets, respectively (ratio Hi-C:shotgun = 0.35).

### Initial processing

We applied bbduk from the BBTools suite (v37.25)^70^ to thoroughly clean raw WGS and Hi-C read libraries (Supplementary Note 7). Processed shotgun reads were assembled into contigs using MEGAHIT (v1.2.9)^13^ with options ‘-min-contig-len 1000 -k-min 21 -k-max 141 -k-step 12 -merge-level 20, 0.95’ (Supplementary Table 9). Then, processed Hi-C paired-end reads were mapped to assembled contigs by BWA MEM (v0.7.17)^71^ with parameter ‘-5SP’. After the alignment, we removed unmapped reads, secondary alignments, supplementary alignments and alignments with low quality (mapping score or nucleotide match length <30). Raw Hi-C contact maps between two contigs were constructed by counting the number of Hi-C read pairs separately aligned to these two contigs.

### Viral contig detection

Long contigs (≥ 3 kbp) assembled from shotgun reads were screened by VirSorter (v1.0.6)^15^ with default parameter to identify viral contigs. VirSorter achieved the best F1 score in a recent benchmarking study^72^. Contigs annotated as prophages were removed from the viral sequences (Supplementary Table 10). We refer to the contigs that are not identified by VirSorter as potential host contigs.

### Construct the Hi-C interaction graph for viral contigs

We define the Hi-C interaction graph for viral contigs as ${{{{{{{{\mathcal{G}}}}}}}}}_{{{{{{{{\rm{hic}}}}}}}}}(V,{E}_{{{{{{{{\rm{hic}}}}}}}}})$, where the vertex ${v}_{i}\in {{{{{{{\mathcal{V}}}}}}}}$ represents the *i*-th identified viral contig, and an edge ${e}_{ij}\in {{{{{{{{\mathcal{E}}}}}}}}}_{{{{{{{{\rm{hic}}}}}}}}}$ exists if *v*_*i*_ and *v*_*j*_ are linked by at least one Hi-C link.

### Construct the host proximity graph for viral contigs

Besides the Hi-C interaction graph, we also take advantage of virus-host proximity structure to link viral contigs. Specifically, we define two viral contigs as associated by *k* shared host contigs if these two viral contigs are linked to at least the same *k* host contigs by the Hi-C interaction. Based on this metric to measure the linkage between viral contigs, we construct the host proximity graph for viral contigs, denoted by ${{{{{{{{\mathcal{G}}}}}}}}}_{{{{{{{{\rm{host}}}}}}}}}(V,{E}_{{{{{{{{\rm{host}}}}}}}}})$, where the vertex ${v}_{i}\in {{{{{{{\mathcal{V}}}}}}}}$ still represents the *i*-th identified viral contig while an edge *e*_*ij*_ exists in ${{{{{{{{\mathcal{E}}}}}}}}}_{{{{{{{{\rm{host}}}}}}}}}$ if *v*_*i*_ and *v*_*j*_ are associated by *k* shared host contigs. Formally, let *H*_*i*_ denote the set of host contigs for viral contig *v*_*i*_. Then, *v*_*i*_ and *v*_*j*_ are connected in the host proximity graph ${{{{{{{{\mathcal{G}}}}}}}}}_{{{{{{{{\rm{host}}}}}}}}}$ if

1 $$\left|{H}_{i}\cap {H}_{j}\right|\ge k,$$

where $\left|\cdot \right|$ denotes the cardinality of a set and the parameter *k* here is automatically determined such that

2 $$\begin{array}{ll}&\mathop{\max }\limits_{k}\,\left|{{{{{{{{\mathcal{E}}}}}}}}}_{{{{{{{{\rm{host}}}}}}}}}\right|\\ &s.t.\,\left|{{{{{{{{\mathcal{E}}}}}}}}}_{{{{{{{{\rm{host}}}}}}}}}\right|\le \left|{{{{{{{{\mathcal{E}}}}}}}}}_{{{{{{{{\rm{hic}}}}}}}}}\right|;\ \,k\ge {k}_{\min },\end{array}$$

where ${k}_{\min }$ (default 4) is the lower bound of parameter *k*. Note that decreasing *k* relaxes the requirement for the existence of an association by shared host contigs, leading to more edges in ${{{{{{{{\mathcal{G}}}}}}}}}_{{{{{{{{\rm{host}}}}}}}}}$. Thus in formula (2), maximizing the number of edges in ${{{{{{{{\mathcal{G}}}}}}}}}_{{{{{{{{\rm{host}}}}}}}}}$ is equivalent to minimize the value of *k*. Though smaller *k* provides a larger number of connections for viral contigs in ${{{{{{{{\mathcal{G}}}}}}}}}_{{{{{{{{\rm{host}}}}}}}}}$, the value of *k* cannot be too small, which may introduce false positive associations due to the experimental noise. Therefore, two constraints that the number of edges in ${{{{{{{{\mathcal{G}}}}}}}}}_{{{{{{{{\rm{host}}}}}}}}}$ is less than or equal to that in ${{{{{{{{\mathcal{G}}}}}}}}}_{{{{{{{{\rm{hic}}}}}}}}}$ and *k* is no less than ${k}_{\min }$ are utilized to control the value of *k*. We found that the vast majority of edges within the host proximity graph linked the viral contigs from the same genome on the three mock metagenomic Hi-C datasets, demonstrating the reliability of the host proximity graph (see Results).

### Integrate the Hi-C interaction graph and the host proximity graph

We have constructed the Hi-C interaction graph and the host proximity graph to link viral contigs. Then, we would like to integrate these two graphs. Let ${{{{{{{{\mathcal{G}}}}}}}}}_{{{{{{{{\rm{int}}}}}}}}}(V,\,{E}_{{{{{{{{\rm{int}}}}}}}}})$ denote the final integrative graph, where the vertex set still represents all viral contigs and an edge *e*_*ij*_ belongs to the edge set ${{{{{{{{\mathcal{E}}}}}}}}}_{{{{{{{{\rm{int}}}}}}}}}$ if *v*_*i*_ and *v*_*j*_ are linked through any one of the Hi-C interaction graph ${{{{{{{{\mathcal{G}}}}}}}}}_{{{{{{{{\rm{hic}}}}}}}}}$ or the host proximity graph ${{{{{{{{\mathcal{G}}}}}}}}}_{{{{{{{{\rm{host}}}}}}}}}$.

### Leiden graph clustering based on the integrative graph

We cluster the viral contigs using the Leiden graph clustering algorithm^46^ based on the integrative graph ${{{{{{{{\mathcal{G}}}}}}}}}_{{{{{{{{\rm{int}}}}}}}}}$. The Leiden algorithm is a modularity-based community detection algorithm. It takes a three-stage greedy approach to optimizing the modularity function. Specifically, in each iteration, the algorithm assigns each node to a community such that the modularity function will increase after the local movement, followed by refining the partition into sub-communities and aggregating the network. Moreover, a general modularity function based on the Reichardt and Bornholdt’s Potts model^73^ is selected for the Leiden algorithm to overcome the resolution limit^74^ and is defined as:

3 $$\mathop{\sum}\limits_{\{i,j|{c}_{i}={c}_{j}\}}({M}_{ij}-r\frac{{d}_{i}{d}_{j}}{2n}),$$

where *M* is the adjacency matrix of graph ${{{{{{{{\mathcal{G}}}}}}}}}_{{{{{{{{\rm{int}}}}}}}}}$, *c* denotes the community of viral vertices, *r* is a resolution parameter, *d* represents the degree of viral vertices and *n* is the total number of edges in graph. The resolution parameter *r* is tuned using the silhouette coefficient^75^ of the binning results, which is a popular clustering evaluation metric without true labels by measuring the cohesion and the separation of the clusters. The candidate resolution that yields the highest silhouette coefficient is selected as the optimal value for the Leiden clustering.

### Evaluate the CheckV completeness of vMAGs on real metagenomic Hi-C datasets

We used one popular tool CheckV (v0.7.0)^43^ to estimate the completeness quality of viral MAGs recovered from three real metagenomic Hi-C datasets. Since CheckV was originally designed for assessing the quality of single-contig viral genomes, viral contigs from each vMAG were concatenated into a single sequence as required by CheckV. CheckV applies two algorithms to compute the completeness of vMAGs based on amino acid identity (AAI) or hidden Markov model (HMM) (Supplementary Note 8). The AAI-based approach reports a confidence level of estimation based on the alignment quality to the CheckV genome database and the contig length, and high- and medium-confidence estimates are demonstrated to be accurate and can be trusted^43^. Therefore, we combined the results estimated by two approaches to determine the completeness of vMAGs. Specifically, for each vMAG, CheckV AAI-based estimation of completeness was utilized if this estimation was qualified as medium or high confidence. Otherwise, the HMM-based estimate was used if available.

### A systematic benchmarking strategy to evaluate the performance of binning viral contigs

#### Rationale of the benchmarking framework

Though CheckV has been widely exploited to evaluate the binning performance for viral contigs, the inability to assess the contamination renders the CheckV evaluation less comprehensive on vMAGs. Moreover, benchmarking the viral genome retrieval through simulation is challenging since few studies have been conducted on modeling Hi-C interactions for viral contigs. To solve these problems, we put forward a benchmarking strategy to comprehensively evaluate the binning performance of Hi-C-based tools on viral contigs without the need for simulating Hi-C interactions for viral contigs.

#### Generate mock viral contigs with ground truth in a mock metagenomic Hi-C dataset directly from the real metagenomic Hi-C sample

Instead of simulating viral contigs using known viral reference genomes, we designed a strategy to directly generate mock viral contigs with ground truth from the real metagenomic Hi-C sample. Though viral genome assemblies from shotgun reads are commonly plagued by insufficiently long contigs, there are still a few single contigs that can individually represent the viral genome with relatively high completeness. Therefore, we first applied CheckV to all identified viral contigs. Contigs above 10,000 bp and marked as ‘high-quality’ or ‘complete’ by CheckV were considered relatively complete viral genomes and served as the putative reference genomes. Then, we directly simulated mock viral contigs from real metagenomic Hi-C datasets using these putative reference genomes. Specifically, we extracted subsequences from putative reference genomes in sliding windows of a 3 kbp length moving from the left to right without overlaps. As a result, putative reference genomes were split into non-overlapping fragments of 3 kbp. Fragments at the edges of putative reference genomes were retained if they were longer than 1 kbp. All fragmented contigs were regarded as mock viral contigs and labeled based on which putative reference genomes they originated from. We then mixed the obtained mock viral contigs with all potential host contigs and aligned the Hi-C read pairs to the mixed contig set using BWA MEM with parameter ‘-5SP’ to create a mock metagenomic Hi-C dataset. In this way, we generated mock viral contigs with ground truth and constructed valid Hi-C interactions without simulating the Hi-C experiments for viral contigs in a mock metagenomic Hi-C dataset. We were subsequently able to validate the binning performance based on mock metagenomic Hi-C datasets for Hi-C-based binning approaches as well as shotgun-based binning tools.

#### Gold standards to evaluate binning performance using mock metagenomic Hi-C datasets

Since the true labels of all mock viral contigs in the mock metagenomic Hi-C dataset were known, we employed four comprehensive evaluation metrics of the clustering performance (Supplementary Note 9): Fowlkes-Mallows scores (F-scores), Adjusted Rand Index (ARI), Normalized Mutual Information (NMI), and Homogeneity. These four metrics were used to evaluate binning performance.

Moreover, we defined the completeness and contamination of each vMAG. Specifically, for each vMAG, we summed the lengths of contigs from different reference genomes separately and assigned the vMAG to the reference genome with the largest query length, denoted by *L*(*q*). We also denoted the length of corresponding reference genome as *L*(*r*) and referred to the total length of the vMAG as *L*(*v*). The completeness of a vMAG is defined as $\frac{L(q)}{L(r)}$ and the contamination of a vMAG is defined as $\frac{L(v)-L(q)}{L(v)}$. Then, we assigned the high-quality vMAGs into three ranks, i.e., near-complete (completeness ≥ 90%, contamination ≤ 10%), substantially complete (70% ≤ completeness <90%, contamination ≤ 10%), and moderately complete (50% ≤ completeness <70%, contamination ≤ 10%), which is similar to the CheckM evaluation criteria^44^.

### The quality control of metagenomic Hi-C datasets

As in^36^, we defined the inter-contig Hi-C contacts as the paired-end Hi-C reads mapped to different viral contigs. Then the 3D ratio was calculated by dividing the number of inter-contig Hi-C contacts by the total number of paired-end Hi-C reads aligned to viral contigs. We also performed an additional quality control step on processed paired-end Hi-C reads using qc3C (v0.5)^76^ in k-mer mode with default parameters. We defined the qc3C CI as the 95% confidence interval of the proportion of observed junction sequences considered to be the product of proximity ligation estimated by the qc3C software. Detailed results of qc3C for each dataset were listed in Supplementary Data 1.

### Annotate vMAGs at the order and family levels

We first employed DemoVir (https://github.com/feargalr/Demovir) to classify viral contigs to the order and family taxonomic levels by comparing genes on contigs against the curated viral protein database (https://figshare.com/articles/NR_Viral_TrEMBL/5822166). Contigs whose genes were consistently classified to the same family were finally annotated. Then, we defined the vMAG family as the family to which the majority of contigs in the vMAG belonged.

### Detect virus-host pairs between vMAGs and host MAGs

All non-viral contigs for each sample were binned using HiCBin (v1.1.0)^40^ with default parameters to generate potential host MAGs, which were subsequently annotated by GTDB-TK (v2.1.0, Release: R207_v2)^54^ with default parameters and the taxonomic classification results were visualized using ITOL (v5)^55^. vMAGs were associated with potential host MAGs if they were linked by at least two Hi-C read-pairs as in^77^.

### Additional validation of ViralCC on a meta 3C/Hi-C dataset

Unlike widely used metagenomic Hi-C technique combining shotgun sequencing with Hi-C sequencing^34,57,67,69,77^, Marbouty et al.^35^ showed that meta3C, another proximity ligation-based approach, allowed the assembly and scaffolding and thus utilized meta3C reads rather than shotgun reads to assemble contigs, which were subsequently linked by Hi-C paired-end reads in their recent experiments on human gut samples^36^. We refer to such datasets as meta 3C/Hi-C datasets. We further validated ViralCC on a meta 3C/Hi-C sample from the human gut microbiome, which consisted of one meta3C library (NCBI accession: SRR11853875) and two separate Hi-C libraries (NCBI accession: SRR13435230 and SRR13435231). Considering the short length of Hi-C reads (35 bp), we neither discarded any Hi-C reads using minimal length option of bbduk^70^ nor trimmed Hi-C reads during the read cleaning step. More details of data processing and the results of validation were shown in Supplementary Note 10.

### Compare ViralCC to other pipelines

VAMB (v3.0.3)^26^ was executed with option ‘-t 40’. vRhyme (v1.0.0)^30^, MetaTOR (v1.1.4)^38^, and bin3C (v0.1.1)^39^ were run with default parameters. The input coverage files of viral contigs for VAMB and vRhyme were generated using script ‘jgi_summarize_bam_contig_depths’ provided by MetaBAT2 (v2.12.1)^25^. Since CoCoNet^29^ removed contigs occurring in only one sample, we used the mode ‘composition’ to recover the viral genomes. The other parameters were set to default values.

### Statistics & Reproducibility

No statistical method was used to predetermine sample size. No data were excluded from the analyses. The experiments were not randomized except for the random binning model where configuration graphs were constructed by randomly assigning edges to match the degree sequence of viral contigs in integrative graphs. The investigators were not blinded to allocation during experiments and outcome assessment.

### Reporting summary

Further information on research design is available in the Nature Portfolio Reporting Summary linked to this article.

## Supplementary information

Supplementary Information

Description of Additional Supplementary Files

Supplementary Data 1

Reporting Summary

## Data Availability

All the datasets used in this study are publicly available from the NCBI Sequence Read Archive database (http://www.ncbi.nlm.nih.gov/sra). The human gut dataset is available under accession codes: shotgun library SRR6131123, Hi-C libraries SRR6131122 and SRR6131124. The cow fecal dataset used in this study is under accession codes: shotgun library ERX2333418, Hi-C libraries ERX2548555 and ERX2548556. The wastewater dataset is available under accession codes: shotgun library SRR8239393 and Hi-C library SRR8239392. The meta 3C/Hi-C dataset used in this study is available under accession codes: meta3C library SRR11853875, Hi-C libraries SRR13435230 and SRR13435231. The databases required by VirSorter can be downloaded at https://zenodo.org/record/1168727/files/virsorter-data-v2.tar.gz. The CheckV reference database is available at https://portal.nersc.gov/CheckV/checkv-db-v1.0.tar.gz. The GTDB-TK reference database can be downloaded at https://data.gtdb.ecogenomic.org/releases/latest/auxillary_files/gtdbtk_v2_data.tar.gz. The curated viral protein database for DemoVir is available at https://figshare.com/articles/NRViralTrEMBL/5822166. The remaining data are available within the Article, Supplementary Information, or Source data. Source data are provided with this paper.

## Source data

Source Data

## Code availability

The ViralCC software is freely available at https://github.com/dyxstat/ViralCCunder the GNU General Public License version v3. The ViralCC code used in this work^78^ is also archived on Zenodo under 10.5281/zenodo.7449911. Scripts to process the intermediate data and plot figures of our ViralCC paper are available at https://github.com/dyxstat/Reproduce_ViralCC/tree/main/Scripts.
