# Supplementary material for: ViralCC retrieves complete viral genomes and virus-host pairs from metagenomic Hi-C data
Source: Nat Commun. 2023 Jan 31;14:502. doi: 10.1038/s41467-023-35945-y (PMC9889337; doi:10.1038/s41467-023-35945-y)
Supplement: Supplementary file 1 — Supplementary Information [file 41467_2023_35945_MOESM1_ESM.pdf]

Supplementary Information

ViralCC retrieves complete viral genomes and virus-host pairs from metagenomic Hi-C data

Yuxuan Du[1], Jed A. Fuhrman[2] and Fengzhu Sun *[1]

[1]Department of Quantitative and Computational Biology, University of Southern California, USA
[2]Department of Biological Sciences, University of Southern California, USA

Table of contents

Supplementary Notes 2

Supplementary Tables 7

Supplementary Figures 24

*Corresponding author: fsun@usc.com

Supplementary Notes

Supplementary Note 1: Binning results for the viral contigs on the mock wastewater dataset and the mock cow fecal dataset

Since all viral contigs were labeled in the mock metagenomic Hi-C datasets, we defined the spurious edges in the host proximity graph of ViralCC as the edges that linked two contigs from different putative reference genomes in the host proximity graph. Then we computed the fraction of spurious edges in the host proximity graph by dividing the number of spurious edges by the total number of edges in the host proximity graph for the three mock metagenomic Hi-C datasets. The fraction was 1.5% and 16.5% for the mock wastewater and mock cow fecal datasets, respectively.

ViralCC was then compared to CoCoNet, vRhyme, bin3C, and MetaTOR on the mock wastewater dataset and the mock cow fecal dataset (Supplementary Fig. 2). VAMB failed to bin viral contigs on both mock datasets due to the small number of contigs to train its model. ViralCC also outperformed other binning methods in terms of F-score, ARI, and homogeneity and recovered the most near-complete and high-quality vMAGs on the mock wastewater dataset. For the mock cow fecal dataset, three Hi-C-based binning methods (i.e., bin3C, MetaTOR, and HiCBin) obtained similar binning performance in terms of the four clustering metrics. MetaTOR and ViralCC achieved slightly better performance in term of NMI and homogeneity, respectively. Although MetaTOR retrieved the most high-quality vMAGs, ViralCC reconstructed the most near-complete vMAGs.

Supplementary Note 2: ViralCC performed better in recovering near complete vMAGs from large putative viral genomes on the mock metagenomic Hi-C datasets

Considering that almost all viral contigs have the same length in our mock datasets, the full recovery of a larger putative viral genomes requires a binner to correctly group more viral contigs into a single bin. To explore the ability of different binners to retrieve large viral genomes, we plotted the genome sizes of the near-complete vMAGs recovered by different binners on the three mock metagenomic Hi-C datasets (Supplementary Fig. 3). On both the mock human gut and wastewater datasets, we found that binners apart from MetaTOR and ViralCC could only retrieve near-complete vMAGs with the reference genome sizes below 60,000 bp. MetaTOR and ViralCC could recover near-complete vMAGs with the reference genome sizes between 60,000 bp and 80,000 bp while ViralCC was the only method that could reconstruct near-complete vMAGs with the reference genome sizes above 80,000 bp. As for the mock cow fecal dataset, the largest reference viral genome is 42,500 bp and all binners could retrieve near-complete vMAGs with the reference genome sizes above 40,000 bp while ViralCC could achieve the largest number. Moreover, we divided the total sizes of near-complete vMAGs by the total sizes of the overall putative viral genomes for the three mock datasets. As shown in Supplementary Table 11, ViralCC achieved the highest genome size fractions of the near-complete vMAGs on all mock datasets. Therefore, ViralCC outperformed other binners on recovering near-complete vMAGs from large putative viral genomes on the mock datasets.

Supplementary Note 3: ViralCC outperformed the random binning according to the CheckV completeness criteria

To construct a random binning model as control experiments, we generated the configuration random graph based on the integrative graph by randomly assigning edges to match the degree sequence of viral contigs in the integrative graph, followed by the Leiden clustering. Since we found

that the silhouette coefficient almost increased monotonically with the increase of the resolution parameter on our random graphs, we run the Leiden algorithm with default parameters and vMAGs were assessed using CheckV. We conducted five rounds of simulations of the random binning on each dataset. As shown in Supplementary Tables 12 to 14, ViralCC markedly outperformed the random control according to the CheckV completeness criteria.

Supplementary Note 4: Results of virus-host detection on the human gut sample

A total of 338 MAGs were generated for non-viral contigs and 164 MAGs could be annotated by GTDB-TK. The CheckM assessment results of 338 MAGs were shown in Supplementary Table 8. *Lachnospirales*, *Oscillospirales*, and *Bacteroidales* are the predominant orders in the human gut sample (Supplementary Table 15).

We then explored the infection spectrum of vMAGs on hosts from different orders (Supplementary Fig. 4). vMAGs from the *Siphoviridae* and *Myoviridae* families were mainly associated with host MAGs from the orders *Lachnospirales* and *Oscillospirales*, followed by *Bacteroidales*. The vast majority of vMAGs from the families *Podoviridae* and *Herpesviridae* were associated with host MAGs from the orders *Lachnospirales* and *Oscillospirales*. CRISPR spacer analysis predicted four interactions between vMAGs and host MAGs, and 3 out of 4 interactions could also be discovered using Hi-C.

Supplementary Note 5: Results of virus-host detection on the cow fecal sample

HiCBin retrieved 496 MAGs for non-viral contigs, which were subsequently evaluated by CheckM (Supplementary Table 8). A total of 191 MAGs could be annotated by GTDB-TK. *Bacteroidales* and *Lachnospirales* are the predominant orders in the cow fecal sample (Supplementary Table 16).

Supplementary Fig. 5 showed the infection spectrum of vMAGs on hosts from different orders. vMAGs from the families *Siphoviridae* and *Myoviridae* were mainly associated with host MAGs from the orders *Bacteroidales* and *Lachnospirales*, followed by *Oscillospirales*, *Coriobacteriales*, and *Erysipelotrichales*. The vast majority of vMAGs from the families *Podoviridae* and *Herpesviridae* were associated with host MAGs from the orders *Bacteroidales* and *Lachnospirales*. CRISPR spacer analysis predicted two interactions between vMAGs and host MAGs. Both interactions could also be discovered using Hi-C.

Supplementary Note 6: The existence of biases by the viral genome sizes in the benchmarking method

According to the benchmarking strategy, we selected the near-complete viral contigs according to the CheckV completeness criteria as putative reference viral genomes. Considering the limitation of the shotgun sequencing, the larger the size of viral genome is, the more difficult it is to recover the complete viral genome using a single contig during the assembly process. Therefore, it is inevitable that the sizes of putative viral genomes tend to be small. Specifically, we found that the sizes of the largest vMAGs were 307,395 bp, 157,462 bp, and 461,626 bp on the human gut, cow fecal, and wastewater datasets, respectively. In contrast, the sizes of the largest selected viral genomes were only 194,784 bp, 42,500 bp, and 127,910 bp for the three datasets, respectively, indicating that the benchmarking method was biased by the viral genome sizes.

Supplementary Note 7: Read cleaning procedure

Adaptor sequences were removed by bbduk from the BBTools suite (v37.25) with parameters 'ktrim=r k=23 mink=11 hdist=1 minlen=50 tpe tbo' and reads were quality-trimmed using bbduk with parameters 'trimq=10 qtrim=r ftm=5 minlen=50'. Then, the first 10 nucleotides of each read were trimmed by bbduk with parameter 'ftl=10'. Identical PCR optical and tile-edge duplicates for Hi-C paired-end reads were removed by the script 'clumpify.sh' from the BBTools suite.

Supplementary Note 8: Algorithms to evaluate the completeness of vMAGs by CheckV

CheckV applies two algorithms to compute the completeness of vMAGs: amino acid identity (AAI)-based and hidden Markov model (HMM)-based approaches. In the AAI-based approach, proteins are first compared to the CheckV genome database using AAI. After identifying the top hits, completeness is computed as the ratio between the contig length and the length of matched reference genomes and a confidence level is reported based on the strength of the alignment and the length of the contig. High- and medium-confidence estimates are quite accurate and can be trusted. The second method is HMM-based. This method aims to compute the completeness of highly novel viruses that may not match a CheckV genome with sufficiently high AAI. In these cases CheckV identifies the viral HMMs on the contig and compares the contig length with reference genomes sharing the same HMMs. CheckV then returns the estimated range for genome completeness, which represents the 90% confidence interval based on the distribution of lengths of reference genomes with the same viral HMMs.

Supplementary Note 9: Evaluation criteria of the clustering results

The Homogeneity Score: Let $C = \{c_i | i = 1, \cdots, n\}$ denote a set of classes and $K = \{k_i | 1, \cdots, m\}$ denote a set of clusters for N samples. Let A be the contingency table produced by the clustering algorithm representing the clustering solution, such that $A = \{a_{ij}\}$ where a_{ij} is the number of data points that are members of class c_i and elements of cluster k_j. Then the homogeneity score, denoted by h is defined as:

$$h = 1 - \frac{H(C|K)}{H(C)}, \tag{S1}$$

where

$$H(C|K) = -\sum_{k=1}^{|K|}\sum_{c=1}^{|C|} \frac{a_{ck}}{N} \log \frac{a_{ck}}{\sum_{c=1}^{|C|} a_{ck}},$$
$$H(C) = -\sum_{c=1}^{|C|} \frac{\sum_{k=1}^{|K|} a_{ck}}{n} \log \frac{\sum_{k=1}^{|K|} a_{ck}}{n}.$$

The Fowlkes-Mallows score: The Fowlkes-Mallows score (F-score) is defined as the geometric mean of the precision and recall, i.e,

$$\text{FM} = \sqrt{\frac{TP}{TP + FP} \cdot \frac{TP}{TP + FN}}, \tag{S2}$$

where TP is the number of true positives, FP is the number of false positives, and FN is the number of false negatives.

The Adjusted Rand Index: The rand index (RI) is defined as the percentage of correct decisions made by the clustering algorithm, i.e.,

$$\mathrm{RI} = \frac{TP + TN}{TP + TN + FP + FN}, \tag{S3}$$

where TP is the number of true positives, TN is the number of true negatives, FP is the number of false positives, and FN is the number of false negatives.

Then, the Adjusted Rand Index (ARI) can be defined as

$$\mathrm{ARI} = \frac{\mathrm{RI} - \mathbb{E}(\mathrm{RI})}{\max(\mathrm{RI}) - \mathbb{E}(\mathrm{RI})}, \tag{S4}$$

where $\mathbb{E}(\mathrm{RI})$ denotes the Expected Rand Index.

The Normalized Mutual Information: Let U and V denote the sets of true class labels and predicted cluster labels, respectively. Define the entropy of a label set S as

$$H(S) = -\sum_{i=1}^{|S|} P(i)\log(P(i)), \tag{S5}$$

where $P(i) = |S_i|/N$ is the probability of an object in class S_i.

The mutual information (MI) between U and V is calculated by:

$$\mathrm{MI}(U, V) = \sum_{i=1}^{|U|}\sum_{j=1}^{|V|} P(i, j)\log(\frac{P(i, j)}{P(i)P'(j)}), \tag{S6}$$

where $P(i, j) = |U_i \cap V_j|/N$, $P(i) = |U_i|/N$, and $P'(j) = |V_j|/N$.

Then, the Normalized Mutual Information (NMI) is defined as

$$\mathrm{NMI}(U, V) = \frac{2 \times \mathrm{MI}(U, V)}{H(U) + H(V)}. \tag{S7}$$

Supplementary Note 10: ViralCC outperformed other binners on a meta 3C/Hi-C dataset derived from a human gut sample

This meta 3C/Hi-C sample was derived from the human gut microbiome and consisted of one meta3C library (NCBI accession: SRR11853875) and two separate Hi-C libraries (NCBI accession: SRR13435230 and SRR13435231). There were 266.5 million meta3C read pairs while the two Hi-C libraries contained 54.9 million (MluCI library) and 56.6 million (HpaII library) read pairs, respectively. Since meta3C reads allowed the assembly and scaffolding, we applied the same initial processing procedure on the raw meta3C reads as the raw shotgun reads to assemble the contigs. A total of 75,745 contigs with length at least 1,000 bp were assembled (N50: 14,449 bp; average length: 5,255 bp; total length: 398,079,188 bp). For the raw Hi-C reads, considering the short length of Hi-C reads, we neither discarded any Hi-C reads using the minimal length option nor trimmed the first ten nucleotides of Hi-C reads during the read cleaning step. Specifically, adaptor sequences of Hi-C reads were removed by bbduk from the BBTools suite (v37.25) with parameters 'ktrim=r

k=23 mink=11 hdist=1 tpe tbo' and reads were quality-trimmed using bbduk with parameters 'trimq=10 qtrim=r ftm=5'. Identical PCR optical and tile-edge duplicates for Hi-C paired-end reads were removed by the script 'clumpify.sh' from the BBTools suite. The alignment of processed Hi-C reads on contigs and the viral contig detection were the same as described in the main text. As a result, 519 contigs were detected as viral contigs by VirSorter.

Viral contigs were binned using different methods on the meta 3C/Hi-C dataset. The CheckV completeness of viral bins was estimated to evaluate the binning quality. We referred to viral bins with CheckV completeness above 90% as draft viral genomes with high completion and denoted bins with CheckV completeness above 50% as draft viral genomes with medium completion. Notably, VAMB failed to bin viral contigs on the meta 3C/Hi-C dataset due to the small number of contigs to train its model. As shown in Supplementary Fig. 6, CoCoNet, vRhyme, bin3C, and MetaTOR could recover 16, 35, 36, and 29 draft viral genomes with high completion, respectively, while ViralCC increased this number to 54. Moreover, CoCoNet, vRhyme, bin3C, and MetaTOR retrieved 19, 53, 44, and 37 draft viral genomes with medium completion, respectively, which was improved to 87 by ViralCC.

Moreover, following Fig. 4 in the main text, we sorted the vMAGs and plotted the raw Hi-C contact maps of the top ten vMAGs for the meta 3C/Hi-C dataset with either the contig index or the contig size as the axis unit (Supplementary Fig. 7), respectively, which confirmed the valid reconstruction of the viral genomes. The specific number of viral contigs and the bin size of these ten vMAGs were shown in Supplementary Table 17.

Finally, 112 vMAGs retrieved by ViralCC could be annotated at the family level on the meta3C/Hi-C dataset. Details of the annotation procedure were described in the main text. Among these 112 vMAGs, 102 (91.1%) vMAGs contained only viral contigs from the same family, demonstrating the high purity of ViralCC's vMAGs at the family level. As shown in Supplementary Fig. 8, bacteriophage *Siphoviridae* was the dominant family, which was consistent with the results of another human gut dataset shown in Fig. 5 in the main text.

Supplementary Tables

Supplementary Table 1. The numbers of putative reference genomes and mock viral contigs from the three metagenomic datasets.

Dataset	Number of putative reference genomes	Number of mock viral contigs
Human gut	51	1,010
Cow fecal	11	94
Wastewater	17	279

Supplementary Table 2. $\mathcal{G}_{\text{int}}$ outperforms $\mathcal{G}_{\text{hic}}$ and $\mathcal{G}_{\text{host}}$ for clustering viral contigs in terms of F-score, ARI, NMI, and homogeneity on the mock human gut dataset.

	F-score	ARI	NMI	Homogeneity
$\mathcal{G}_{\text{hic}}$	0.491	0.383	0.834	0.717
$\mathcal{G}_{\text{host}}$	0.449	0.335	0.761	0.616
$\mathcal{G}_{\text{int}}$	**0.795**	**0.787**	**0.929**	**0.921**

Note: the optimal values of the results are in **bold**.

Supplementary Table 3. $\mathcal{G}_{\text{int}}$ outperforms $\mathcal{G}_{\text{hic}}$ and $\mathcal{G}_{\text{host}}$ for clustering in terms of the completeness and contamination criteria on the mock human gut dataset.

	near-complete	substantially complete	moderately complete	total
$\mathcal{G}_{\text{hic}}$	8	3	5	16
$\mathcal{G}_{\text{host}}$	12	2	0	14
$\mathcal{G}_{\text{int}}$	26	2	4	32

Supplementary Table 4. The numbers of viral contigs and the bin sizes of the top ten vMAGs retrieved from the human gut dataset.

vMAG index	Number of viral contigs	Bin size (bp)
vMAG 1	10	97,408
vMAG 2	9	144,968
vMAG 3	9	136,680
vMAG 4	9	135,075
vMAG 5	9	123,490
vMAG 6	9	90,209
vMAG 7	9	85,181
vMAG 8	8	136,845
vMAG 9	7	76,540
vMAG 10	7	64,147

Supplementary Table 5. The numbers of viral contigs and the bin sizes of the top ten vMAGs retrieved from the cow fecal dataset.

vMAG index	Number of viral contigs	Bin size (bp)
vMAG 1	15	116,220
vMAG 2	13	113,782
vMAG 3	12	128,510
vMAG 4	11	157,462
vMAG 5	11	85,441
vMAG 6	11	74,779
vMAG 7	11	62,022
vMAG 8	11	60,308
vMAG 9	10	90,396
vMAG 10	10	80,082

Supplementary Table 6. The numbers of viral contigs and the bin sizes of the top ten vMAGs retrieved from the wastewater dataset.

vMAG index	Number of viral contigs	Bin size (bp)
vMAG 1	20	461,626
vMAG 2	20	207,665
vMAG 3	19	117,968
vMAG 4	18	320,758
vMAG 5	18	150,122
vMAG 6	17	184,707
vMAG 7	17	148,972
vMAG 8	17	124,626
vMAG 9	16	187,166
vMAG 10	16	159,152

Supplementary Table 7. The quality control results of the three metagenomic Hi-C datasets.

Dataset	3D ratio	qc3C CI
Human gut	23.3%	[5.933%, 5.943%]
Cow fecal	38.3%	[52.06%, 52.08%]
Wastewater	54.9%	[30.65%, 30.67%]

Note: qc3C CI represents the 95% confidence interval of the proportion of observed junction sequences considered to be the product of proximity ligation estimated by the qc3C software.

Supplementary Table 8. The numbers of high-quality potential host MAGs retrieved by HiCBin from the three metagenomic Hi-C datasets according to the CheckM criteria.

Dataset	Near-complete	Substantially complete	Moderately complete
Human gut	67	33	12
Cow fecal	79	27	17
Wastewater	94	56	41

Note: near-complete (CheckM completeness $\geq$ 90%, CheckM contamination $\leq$ 10%), substantially complete (70% $\leq$ CheckM completeness <90%, CheckM contamination $\leq$ 10%), and moderately complete (50% $\leq$ CheckM completeness <70%, CheckM contamination $\leq$ 10%)

Supplementary Table 9. The statistics of assembled contigs for the three metagenomic Hi-C datasets.

Dataset	Contigs $\geq$ 1 kbp	N50	Average length (bp)	Total length (bp)
Human gut	105,267	14,166	5,044	530,969,816
Cow fecal	190,817	5,820	3,573	681,943,699
Wastewater	752,580	2,977	2,538	1,910,562,642

Note: N50 is defined by the length of the shortest contig where contigs with longer and equal length cover at least 50% of the assembly.

Supplementary Table 10. The statistics of viral contigs detected by VirSorter for the three metagenomic Hi-C datasets.

Dataset	Number of viral contigs	N50	Total length
Human gut	791	9,198	13,226,295
Cow fecal	1,338	6,038	12,466,164
Wastewater	2,757	5,960	25,549,304

Note: N50 is defined by the length of the shortest contig where contigs with longer and equal length cover at least 50% of the assembly.

Supplementary Table 11. The genome size fractions of near-complete vMAGs recovered by different binners on the three mock datasets.

	Mock human gut	Mock cow fecal	Mock wastewater
VAMB	1.1%	NA	NA
vRhyme	0	20.4%	11.6%
bin3C	5.1%	44.9%	5.1%
CoCoNet	4.5%	14.9%	13.9%
MetaTOR	35.3%	59.5%	51.8%
ViralCC	**45.6%**	**85.1%**	**65.4%**

Note: VAMB failed to bin viral contigs on the mock wastewater and cow fecal datasets due to the small number of contigs to train its model. The optimal values of the results are in bold.

Supplementary Table 12. The numbers of vMAGs retrieved by configuration random graphs on the human gut dataset according to the CheckV completeness criteria.

CheckV completeness	$\geq$ 90%	$\geq$ 80%	$\geq$ 70%	$\geq$ 60%	$\geq$ 50%	total
Simulation 1	15	1	3	1	3	23
Simulation 2	15	1	3	0	3	22
Simulation 3	14	1	3	0	3	21
Simulation 4	14	1	3	1	3	22
Simulation 5	13	1	3	0	3	20

Supplementary Table 13. The numbers of vMAGs retrieved by configuration random graphs on the cow fecal dataset according to the CheckV completeness criteria.

CheckV completeness	$\geq$ 90%	$\geq$ 80%	$\geq$ 70%	$\geq$ 60%	$\geq$ 50%	total
Simulation 1	14	1	0	2	1	18
Simulation 2	13	1	0	2	1	17
Simulation 3	15	1	0	2	1	19
Simulation 4	15	1	0	2	1	19
Simulation 5	14	1	0	2	1	18

Supplementary Table 14. The numbers of vMAGs retrieved by configuration random graphs on the wastewater dataset according to the CheckV completeness criteria.

CheckV completeness	$\geq 90\%$	$\geq 80\%$	$\geq 70\%$	$\geq 60\%$	$\geq 50\%$	total
Simulation 1	25	3	1	5	3	37
Simulation 2	26	2	1	5	3	37
Simulation 3	24	3	1	5	2	35
Simulation 4	25	2	1	5	2	35
Simulation 5	25	2	1	6	2	36

Supplementary Table 15. The taxonomic statistics of potential host MAGs derived from the human gut dataset.

Order	Number of MAGs
Lachnospirales	62
Oscillospirales	53
Bacteroidales	23
Coriobacteriales	6
Actinomycetales	6
Lactobacillales	4
Christensenellales	2
Peptostreptococcales	2
Clostridiales	2
Erysipelotrichales	2
Veillonellales	1
Peptococcales	1

Supplementary Table 16. The taxonomic statistics of potential host MAGs derived from the cow fecal dataset.

Order	Number of MAGs
Bacteroidales	58
Lachnospirales	49
Oscillospirales	14
Coriobacteriales	10
Peptostreptococcales	8
Erysipelotrichales	8
Selenomonadales	8
Acidaminococcales	7
Enterobacterales	4
Actinomycetales	3
Veillonellales	3
Sphaerochaetales	3
Treponematales	2
Christensenellales	1
Eubacteriales	1
Desulfovibrionales	1
Gastranaerophilales	1

Supplementary Table 17. The numbers of viral contigs and the bin sizes of the top ten vMAGs retrieved from the meta 3C/Hi-C dataset of the human gut microbiome.

vMAG index	Number of viral contigs	Bin size (bp)
vMAG 1	13	146,270
vMAG 2	11	68,170
vMAG 3	10	99,228
vMAG 4	8	222,854
vMAG 5	8	150,466
vMAG 6	8	119,736
vMAG 7	8	95,531
vMAG 8	8	76,880
vMAG 9	7	68,408
vMAG 10	7	45,889

Supplementary Figures

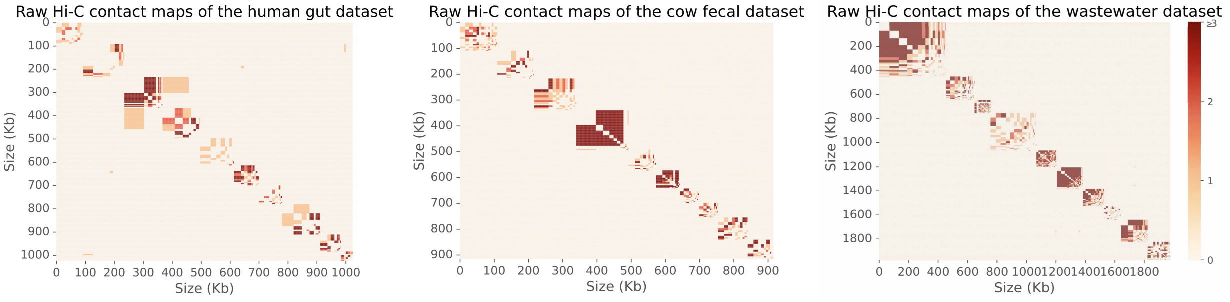

(a) (b) (c)

Supplementary Fig. 1: Heatmaps of raw Hi-C contact matrices of the top ten vMAGs on the (a) human gut, (b) cow fecal, and (c) wastewater datasets with the contig size as the axis unit. The vMAGs were first ranked by their numbers of contigs and then the contigs within each vMAG were ranked by their sizes. The scale bar shows the number of raw Hi-C contacts between viral contigs.

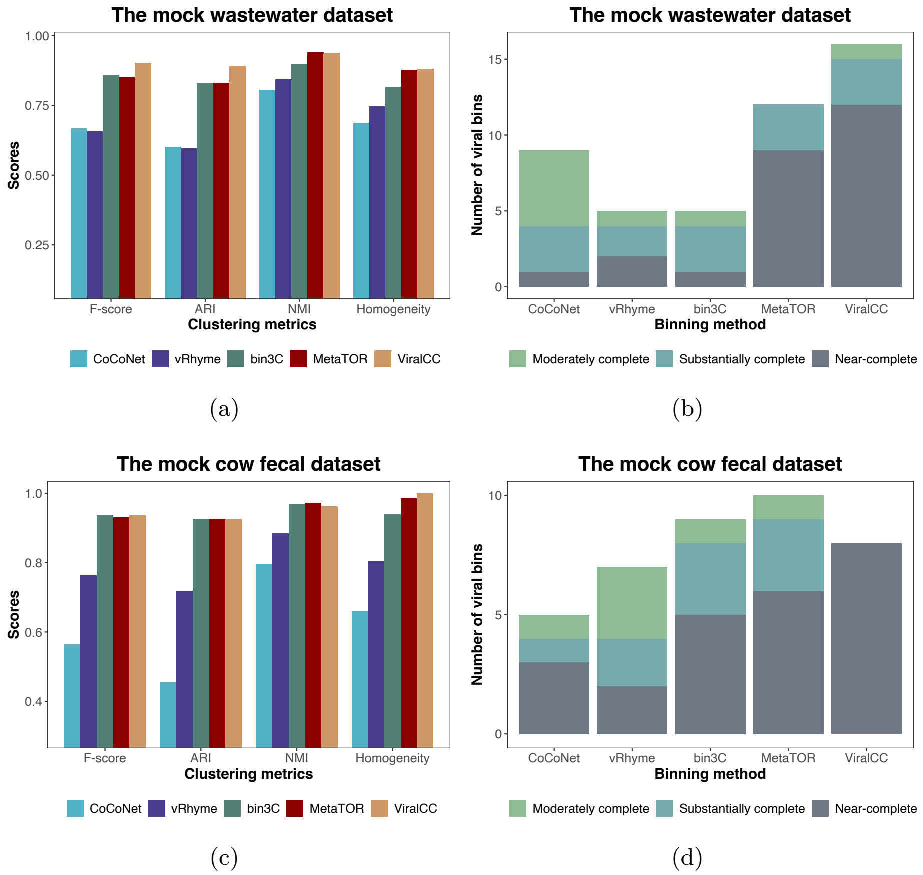

Supplementary Fig. 2: Comparison of viral genome retrieval performance according to the (a) clustering metrics and (b) completeness and contamination criteria on the mock wastewater dataset, and the (c) clustering metrics and (d) completeness and contamination criteria on the mock cow fecal dataset. Moderately complete: $50\% \leq$ completeness $<70\%$, contamination $\leq 10\%$; Substantially complete: $70\% \leq$ completeness $<90\%$, contamination $\leq 10\%$; Near-complete: completeness $\geq 90\%$, contamination $\leq 10\%$.

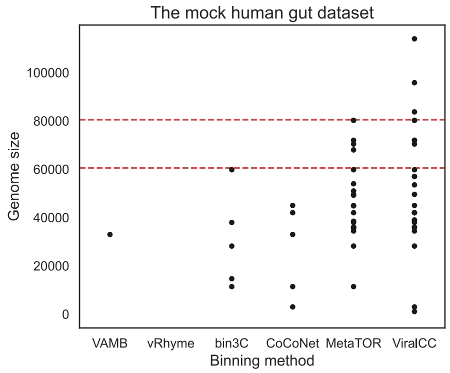

(a)

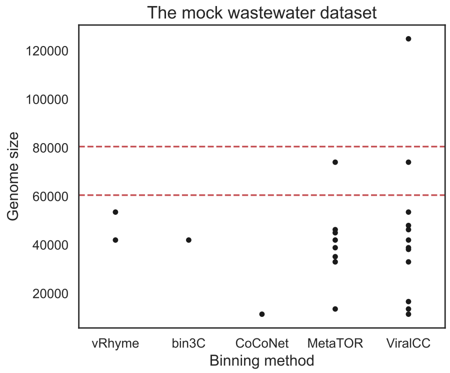

(b)

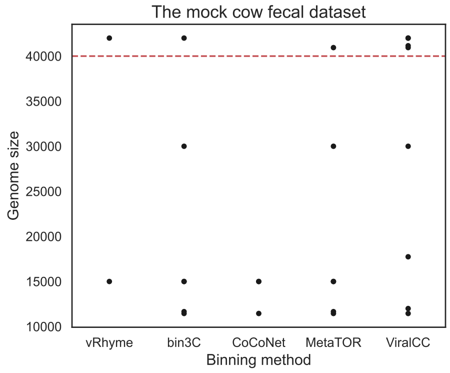

(c)

Supplementary Fig. 3: The strip plot of the genome sizes of the near-complete vMAGs recovered by different binners on the mock (a) human gut, (b) wastewater, and (c) cow fecal datasets. VAMB failed to bin viral contigs on the mock wastewater and cow fecal datasets due to the small number of contigs to train its model.

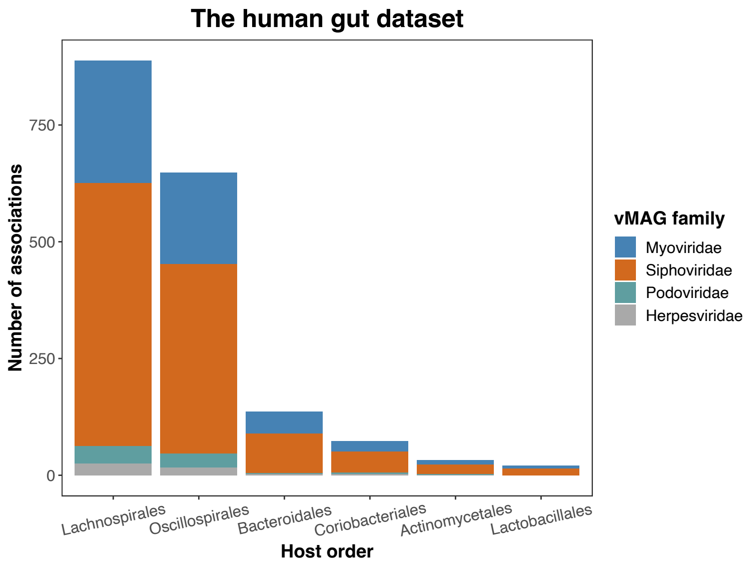

Supplementary Fig. 4: The apparent infection spectrum of vMAGs on hosts from different orders on the human gut dataset.

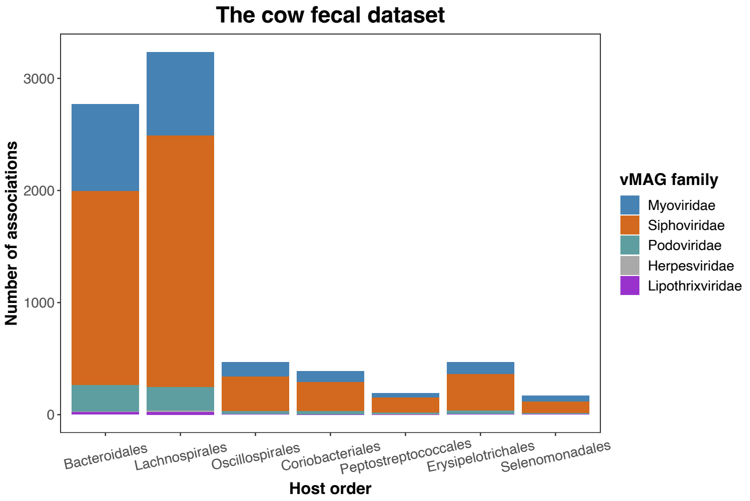

Supplementary Fig. 5: The apparent infection spectrum of vMAGs on hosts from different orders on the cow fecal dataset.

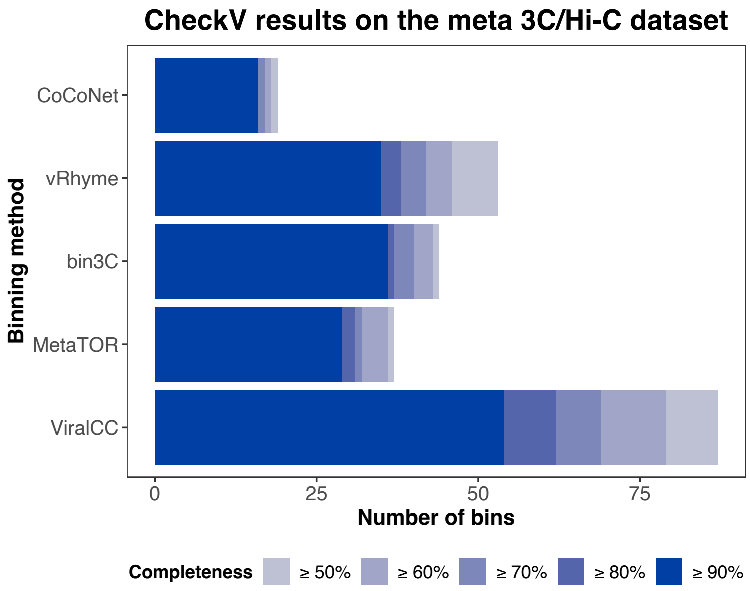

Supplementary Fig. 6: Comparison of draft viral bins retrieved by different binning tools according to the CheckV completeness standard on the meta 3C/Hi-C dataset of the human gut microbiome.

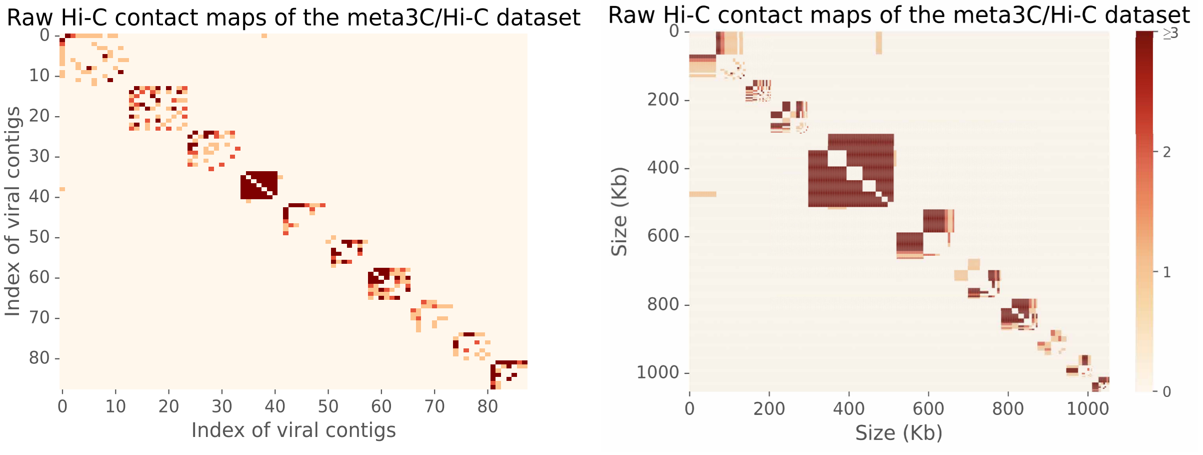

(a) (b)

Supplementary Fig. 7: Heatmaps of raw Hi-C contact matrices of the top ten vMAGs on the meta 3C/Hi-C dataset of the human gut microbiome with (a) the contig index and (b) the contig size as the axis unit. The vMAGs were first ranked by their numbers of contigs and then the contigs within each vMAG were ranked by their sizes. The scale bar shows the number of raw Hi-C contacts between viral contigs.

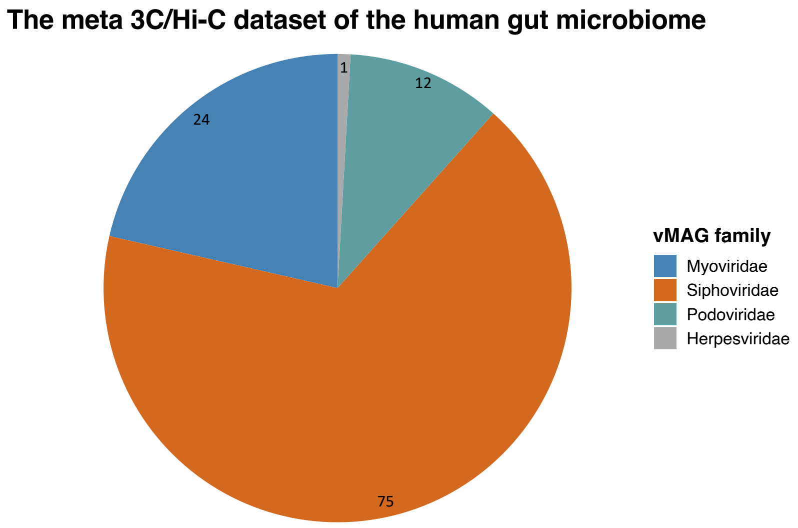

Supplementary Fig. 8: Taxonomy statistics of annotated vMAGs on the meta 3C/Hi-C dataset of the human gut microbiome. The numbers on the graph indicate the number of vMAGs belonging to different families.
