# Supplementary material for: ViralCC retrieves complete viral genomes and virus-host pairs from metagenomic Hi-C data
Source: Nat Commun. 2023 Jan 31;14:502. doi: 10.1038/s41467-023-35945-y (PMC9889337; doi:10.1038/s41467-023-35945-y)
Supplement: Supplementary file 2 — Description of Additional Supplementary Files [file 41467_2023_35945_MOESM2_ESM.pdf]

Description of Additional Supplementary Files

File Name: Supplementary Data 1
Description: Detailed results of qc3C for real metagenomic Hi-C datasets.
