# Supplementary material for: ViralCC retrieves complete viral genomes and virus-host pairs from metagenomic Hi-C data
Source: Nat Commun. 2023 Jan 31;14:502. doi: 10.1038/s41467-023-35945-y (PMC9889337; doi:10.1038/s41467-023-35945-y)
Supplement: Supplementary file 4 — Reporting Summary [file 41467_2023_35945_MOESM4_ESM.pdf]

nature portfolio

Corresponding author(s): Fengzhu Sun

Last updated by author(s): Dec 19, 2022

Reporting Summary

Nature Portfolio wishes to improve the reproducibility of the work that we publish. This form provides structure for consistency and transparency in reporting. For further information on Nature Portfolio policies, see our Editorial Policies and the Editorial Policy Checklist.

Statistics

For all statistical analyses, confirm that the following items are present in the figure legend, table legend, main text, or Methods section.

n/a	Confirmed	
x		The exact sample size (*n*) for each experimental group/condition, given as a discrete number and unit of measurement
x		A statement on whether measurements were taken from distinct samples or whether the same sample was measured repeatedly
x		The statistical test(s) used AND whether they are one- or two-sided<br>*Only common tests should be described solely by name; describe more complex techniques in the Methods section.*
x		A description of all covariates tested
x		A description of any assumptions or corrections, such as tests of normality and adjustment for multiple comparisons
	x	A full description of the statistical parameters including central tendency (e.g. means) or other basic estimates (e.g. regression coefficient) AND variation (e.g. standard deviation) or associated estimates of uncertainty (e.g. confidence intervals)
x		For null hypothesis testing, the test statistic (e.g. *F*, *t*, *r*) with confidence intervals, effect sizes, degrees of freedom and *P* value noted<br>*Give P values as exact values whenever suitable.*
x		For Bayesian analysis, information on the choice of priors and Markov chain Monte Carlo settings
x		For hierarchical and complex designs, identification of the appropriate level for tests and full reporting of outcomes
x		Estimates of effect sizes (e.g. Cohen's *d*, Pearson's *r*), indicating how they were calculated

Our web collection on statistics for biologists contains articles on many of the points above.

Software and code

Policy information about availability of computer code

Data collection	SRA-Toolkit (v2.10.8) was used to collect data from the NCBI database.

Data analysis	ViralCC v1.0.0<br>BBTools v37.25<br>MEGAHIT v1.2.9<br>BWA MEM v0.7.17<br>VirSorter v1.0.6<br>CheckV 0.7.0<br>VAMB v3.0.3<br>CoCoNet v1.1.0<br>vRhyme v1.0.0<br>bin3C v0.1.1<br>MetaBAT2 v2.12.1<br>MetaTOR v1.1.4<br>qc3C v0.5<br>GTDB-TK v2.1.0<br>ITOL v5<br>HiCBin v1.1.0<br>DemoVir https://github.com/feargalr/Demovir

For manuscripts utilizing custom algorithms or software that are central to the research but not yet described in published literature, software must be made available to editors and reviewers. We strongly encourage code deposition in a community repository (e.g. GitHub). See the Nature Portfolio guidelines for submitting code & software for further information.

Data

Policy information about availability of data

All manuscripts must include a data availability statement. This statement should provide the following information, where applicable:

- Accession codes, unique identifiers, or web links for publicly available datasets
- A description of any restrictions on data availability
- For clinical datasets or third party data, please ensure that the statement adheres to our policy

There is no restriction on data availability.

All the datasets used in this study are publicly available from the NCBI Sequence Read Archive database (http://www.ncbi.nlm.nih.gov/sra). The human gut dataset is available under accession codes: shotgun library SRR6131123, Hi-C libraries SRR6131122 and SRR6131124. The cow fecal dataset used in this study is under accession codes: shotgun library ERX2333418, Hi-C libraries ERX2548555 and ERX2548556. The wastewater dataset is available under accession codes: shotgun library SRR8239393 and Hi-C library SRR8239392. The meta 3C/Hi-C dataset used in this study is available under accession codes: meta3C library SRR11853875, Hi-C libraries SRR13435230 and SRR13435231. The databases required by VirSorter can be downloaded at https://zenodo.org/record/1168727/files/virsorter-data-v2.tar.gz. The CheckV reference database is available at https://portal.nersc. gov/CheckV/checkv-db-v1.0.tar.gz. The GTDB-TK reference database can be downloaded at https://data.gtdb.ecogenomic.org/releases/latest/auxillary files/gtdbtk v2 data.tar.gz. The curated viral protein database for DemoVir is available at https://figshare.com/articles/NRViralTrEMBL/5822166. The remaining data are available within the Article, Supplementary Information, or Source data. Source data are provided with this paper.

Human research participants

Policy information about studies involving human research participants and Sex and Gender in Research.

Reporting on sex and gender	This research does not involve human research participants. All human gut data are from publicly available datasets.
Population characteristics	This research does not involve human research participants. All human gut data are from publicly available datasets.
Recruitment	This research does not involve human research participants. All human gut data are from publicly available datasets.
Ethics oversight	This research does not involve human research participants. All human gut data are from publicly available datasets.

Note that full information on the approval of the study protocol must also be provided in the manuscript.

Field-specific reporting

Please select the one below that is the best fit for your research. If you are not sure, read the appropriate sections before making your selection.

[x] Life sciences [ ] Behavioural & social sciences [ ] Ecological, evolutionary & environmental sciences

For a reference copy of the document with all sections, see nature.com/documents/nr-reporting-summary-flat.pdf

Life sciences study design

All studies must disclose on these points even when the disclosure is negative.

Sample size	No sample-size calculation was performed. We used three mock and four real metagenomic Hi-C datasets from the human gut, the cow fecal, and the wastewater environments. The diversity of microbial ecosystems utilized in the benchmarking ensures the sufficiency of the analysis.
Data exclusions	No data were excluded from the analyses.
Replication	No experimental replication was performed except for the random binning model on real metagenomic Hi-C datasets. The random binning were replicated five times and all attempts at replication were successful. The seed for the Leiden clustering was set to ensure the reproducibility of all results shown in the paper.
Randomization	Since each metagenomic Hi-C dataset was derived from one single sample, there was no assignment of samples into experimental groups and there were no covariates across samples in the same dataset. Therefore, randomization was not relevant to sample allocations and covariate controls. In the random binning model, configuration random graphs were constructed by randomly assigning edges to match the degree sequence of viral contigs in integrative graphs.
Blinding	Since each metagenomic Hi-C dataset was derived from one single sample, we neither allocated samples into different experimental groups nor split samples into training and testing. Therefore, the blinding was not relevant to our study.

Reporting for specific materials, systems and methods

We require information from authors about some types of materials, experimental systems and methods used in many studies. Here, indicate whether each material, system or method listed is relevant to your study. If you are not sure if a list item applies to your research, read the appropriate section before selecting a response.

Materials & experimental systems

n/a	Involved in the study	
☒	☐	Antibodies
☒	☐	Eukaryotic cell lines
☒	☐	Palaeontology and archaeology
☒	☐	Animals and other organisms
☒	☐	Clinical data
☒	☐	Dual use research of concern

Methods

n/a	Involved in the study	
☒	☐	ChIP-seq
☒	☐	Flow cytometry
☒	☐	MRI-based neuroimaging
